# Adaptive Feature Fusion Gate and Gated Channel-Spatial Attention in CNN-Transformer Models for Music Genre Classification

**DOI:** 10.1371/journal.pone.0344606

**Published:** 2026-04-09

**Authors:** Yunyan Ma, Zhenwu Ding, Shuang Wan, Hui Li, Yuan Xu

**Affiliations:** 1 School of Music, Jiangxi University of Applied Science and Technology, Nanchang, China; 2 College of Artificial Intelligence, Dongguan City University, Dongguan, China; University of Salerno: Universita degli Studi di Salerno, ITALY

## Abstract

With the rapid growth of music data, automatic music genre classification has become a critical task in music information retrieval. Traditional methods based on handcrafted features are increasingly inadequate when handling large-scale analysis. This paper proposes the Convolutional Neural Network-Gated Transformer Network (CT-GateNet), a hybrid architecture that integrates a gated channel-spatial attention mechanism with an adaptive feature fusion gating mechanism to achieve discriminative feature learning and efficient feature integration. To mitigate data scarcity, a data augmentation strategy based on a denoising diffusion probabilistic model is introduced. Experiments are conducted on three public music genre datasets: GTZAN, FMA-SMALL and FMA-Medium. The method achieves classification accuracies of 98.72%, 89.42%, and 69.07% on GTZAN, FMA-SMALL and FMA-Medium, respectively, demonstrating outstanding performance and robust generalization capabilities. These results validate CT-GateNet’s effectiveness in music genre classification and provide valuable insights for audio classification research.

## 1. Introduction

According to the IFPI Global Music Report 2025, the global recorded music industry grew for the tenth consecutive year, reaching US $29.6 billion in revenues. This exponential growth in content underscores the urgent need for efficient, scalable music organization systems. Historically, music genre classification has relied on handcrafted features combined with traditional machine learning methods. While such approaches reduce modeling complexity to some extent, they suffer from low efficiency and are prone to error when handling large-scale music data [1]. Recent advances in deep learning offer promising alternatives. For example, Ghildiyal et al. [2] introduced two methods for music genre classification: an end-to-end CNN-based spectrogram model and a hybrid method integrating handcrafted features with machine learning classifiers. Their ensemble model attained an AUC of 0.894 on the AudioSet dataset. However, the model employs only a shallow 5-layer CNN, which limits its capacity and fails to address abundant redundant features in spectrograms that hamper classification performance. In another study, Wang et al. [3] proposed the Deformer model, which learns audio representations via a denoising process without complex pre-training. It achieved 84.5% accuracy on GTZAN, surpassing ResNet-BiGRU (81%) and S3T (81.1%). Nonetheless, the model relies solely on Transformers, which excel in global context modeling but struggle to capture localized spectral patterns—such as instrumental textures and harmonic transitions that are critical to discriminating complex musical passages.

In summary, current deep learning approaches still face three core challenges: (1) redundant information in audio data distracts models from learning discriminative features; (2) unimodal architectures (e.g., CNN-only or Transformer-only models) are limited in capturing both local timbral patterns and global temporal dependencies; and (3) effective fusion of multi-level features in hybrid architectures remains an open problem. To address these issues, this paper introduces two key innovations:

- A Gated Channel-Spatial Attention (GCSA) module, employing a dual-path architecture with dynamic feature recalibration to suppress redundant information and enhance discriminative feature learning.
- An Adaptive Feature Fusion Gate (AFFG) mechanism, integrated within a novel CNN-Transformer hybrid framework, to adaptively combine local spectral features and global temporal representations without manual weight tuning.

The remainder of this paper is organized as follows: Section 2 reviews related work; Section 3 details the proposed methodology; Section 4 describes the experimental setup and preparation; Section 5 presents the experimental results and analysis; Section 6 discusses the findings and underlying reasons; and Section 7 concludes the paper and outlines future research directions.

## 2. Related work

Early studies in music classification primarily relied on convolutional neural network architectures to achieve end-to-end feature learning through spectrograms. Research has demonstrated that CNNs can effectively replace traditional handcrafted feature engineering by directly extracting discriminative local patterns from time-frequency representations. However, pure CNN architectures exhibit inherent limitations; their local receptive fields struggle to model long-range temporal dependencies in music signals, leading to constrained classification performance on the GTZAN dataset, as evidenced by study [4] achieving only 70.60% accuracy.

To overcome this bottleneck, subsequent research attempted to incorporate recurrent neural network components to enhance temporal modeling capabilities, yet these approaches encountered challenges such as low training efficiency and gradient instability. In recent years, CNN-Transformer hybrid architectures have emerged as a mainstream research direction. Among them, studies [5] have achieved significant performance improvements on the GTZAN and FMA datasets by synergistically leveraging the local feature extraction capability of CNNs and the global dependency modeling strength of Transformers. Furthermore, innovative methods such as the zero-shot learning framework proposed in [6] and the multi-scale Transformer architecture developed in [7] have further expanded the technical pathways for this task.

## 3. Methodology

As established in the preceding analysis, while Transformer architectures demonstrate exceptional capability in capturing global contextual information, their performance in local feature extraction remains suboptimal with substantial computational demands. Conversely, convolutional neural networks exhibit remarkable proficiency in local pattern recognition but lack the capacity to model long-range dependencies or effectively discriminate between salient and peripheral regions. These complementary characteristics motivate the integration of both architectures into a unified framework.

**Fig 1:**
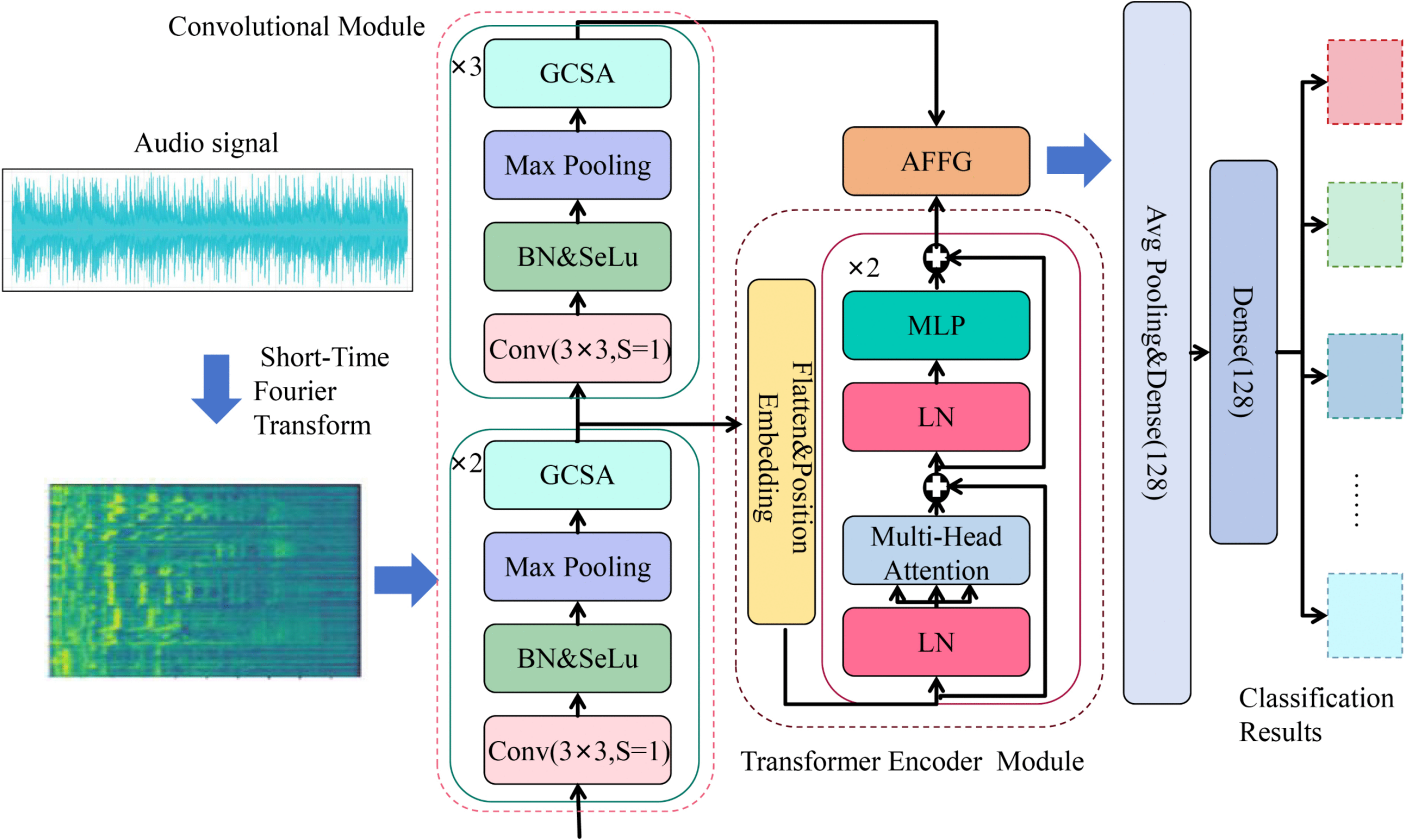
The overall architecture of the proposed CT-GateNet model.

This paper presents CT-GateNet, a novel hybrid architecture that synergistically combines convolutional networks with Transformer encoders for music genre classification. As illustrated in Fig 1, the proposed architecture features a five-stage CNN backbone with progressively expanding filter dimensions (32, 64, 128, 256, 512), where 3×3 convolutions capture fine-grained spectral patterns while dilated convolutions enlarge the receptive field. To address the prevalent issue of feature redundancy in conventional CNN feature extraction, the Gated Channel-Spatial Attention (GCSA) module is proposed, enhancing feature discriminability through parallel dual-path attention mechanisms and dynamic feature calibration. The refined features are subsequently processed by a 2-layer Transformer encoder with 512-dimensional embeddings and multi-head attention to capture global temporal dependencies. Furthermore, an Adaptive Feature Fusion Gate (AFFG) dynamically balances contributions from both pathways through learnable weights, enabling optimal integration of local and global representations. The architecture ultimately comprises a fully connected layer with 128 units (0.45 dropout) and a classification head with softmax outputs, totaling 9 million parameters.

### 3.1 Gated channel-spatial attention

**Fig 2:**
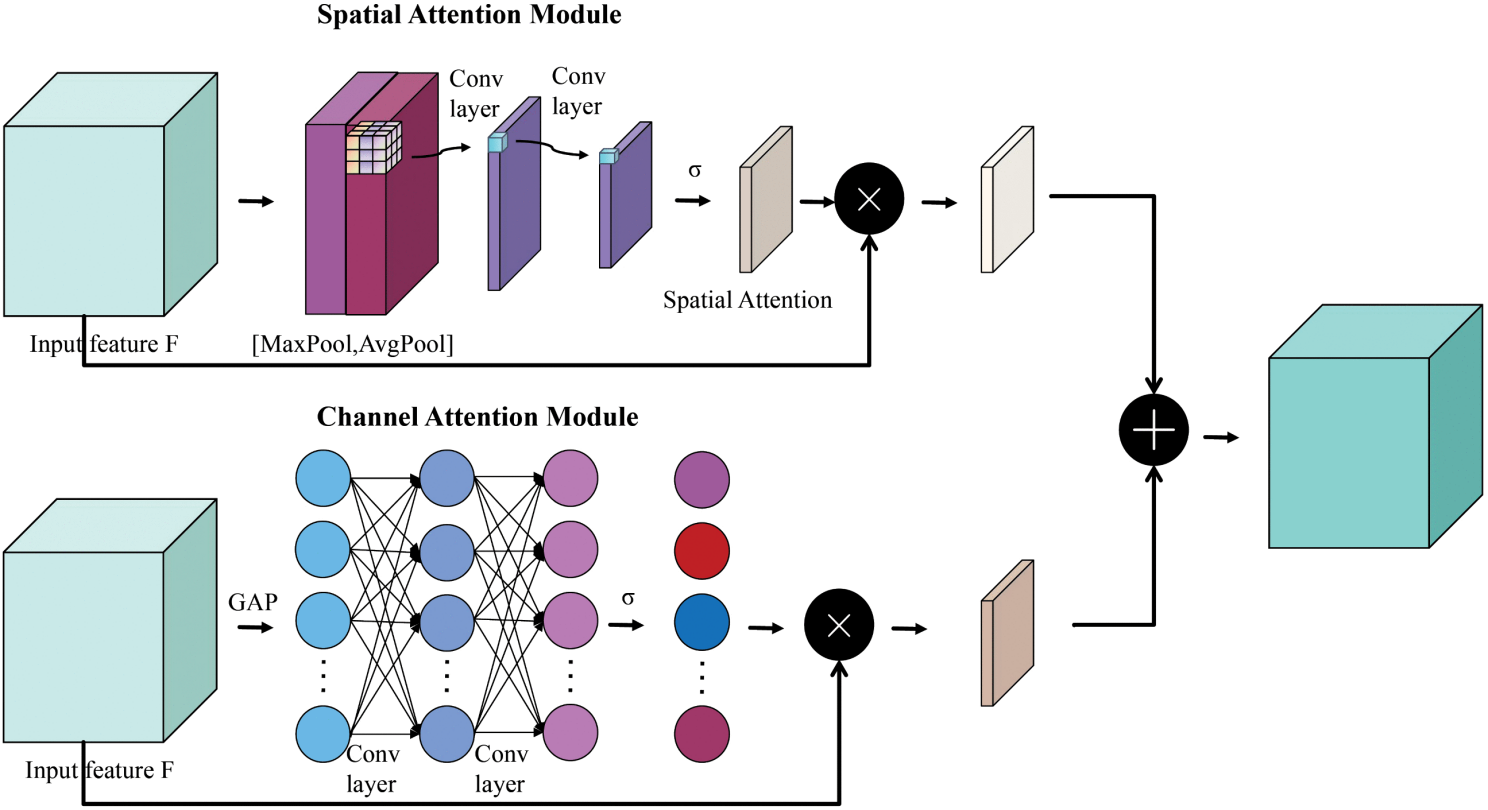
Diagram of our Gated Channel-Spatial Attention (GCSA) mechanism.

In order to enhance the ability to capture detailed feature information in audio features, inspired by ECA-Net [8] and CBAM [9], this paper proposes the Gated Channel-Spatial Attention (GCSA) mechanism. As shown in Fig 2. The GCSA mechanism employs parallel channel attention and spatial attention pathways, coupled with a gating mechanism to dynamically recalibrate feature maps. In the channel attention branch, global average pooling is initially applied to compress the feature maps across the spatial dimensions, thereby retaining the global information among channels. Subsequently, each channel’s importance for the current task is reflected through the weights generated by a fully connected layer and an activation function. The mathematical formulation is presented as follows:


$$z_{c}=\frac{1}{H\times W}\sum_{i=1}^{H}\sum_{j=1}^{W}x_{ij}\;\text{(Global Average Pooling), }$$ (1)

$$w_{c}=\sigma (W_{2}\delta (W_{1}z_{c}))\;\text{(Two-layer MLP). }$$ (2)

where $W_{1}\in {\mathbb{R}}^{C/r\times C}$, $W_{2}\in {\mathbb{R}}^{C\times C/r}$ are learnable weights, *r* = 16 is the reduction ratio, δ denotes the ReLU activation.

The spatial attention branch acquires the maximum and average responses of the feature maps across the spatial dimensions through global maximum pooling and global average pooling, respectively. After concatenating these two results along the channel dimension, a spatial attention weight map is generated via a convolutional layer and an activation function. This weight map assigns weights to each spatial location of the feature maps, thereby highlighting key time frames and frequency bands. The mathematical formulation is presented as follows:


$$z_{\text{avg}}=\text{mean}(x,\text{axis}=-1),$$ (3)

$$z_{\text{max}}=\text{max}(x,\text{axis}=-1),$$ (4)

$$w_{s}=\sigma (f^{7\times 7}([z_{\text{avg}};z_{\text{max}}])).$$ (5)

where $f^{7\times 7}$ represents a 7×7 convolutional kernel, [;] denotes channel concatenation.

Ultimately, the outputs of the channel attention and spatial attention are deeply integrated through element-wise multiplication. This integration not only preserves the significant feature information from both the channel and spatial dimensions but also dynamically adjusts the contribution ratio of the two in response to different input data through the gating mechanism. The gating fusion is formulated as follows:


$$w_{final}=w_{c}⊙w_{s},$$ (6)

$$x_{out}=x⊙w_{final}.$$ (7)

where ⊙ denotes element-wise multiplication. Experimental results demonstrate that this design improves classification accuracy by approximately 1.2% compared to traditional concatenation fusion.

### 3.2 Adaptive feature fusion gate

**Fig 3:**
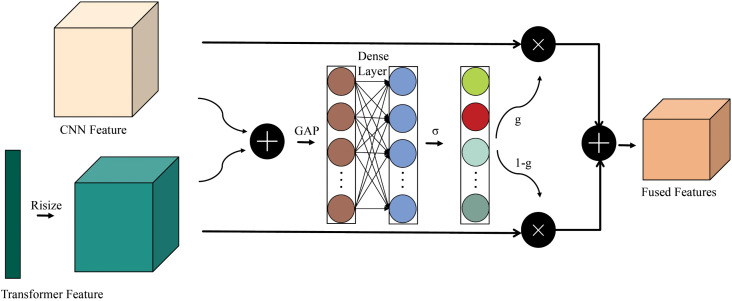
The Adaptive Feature Fusion Gate (AFFG) mechanism.

To address the issue of manually setting weights for feature fusion in CNN-Transformer hybrid architectures, this paper proposes the AFFG mechanism, aiming to fully leverage the respective strengths of CNNs in local feature extraction and Transformers in global feature modeling and achieve adaptive fusion between the two. As shown in Fig 3. Due to the spatial dimension differences between the features output by CNNs and Transformers, bilinear interpolation is first used to adjust the Transformer features to the same spatial dimension as the CNN features, ensuring effective fusion operations can be performed on the same spatial dimension. Then, the aligned CNN features and Transformer features are concatenated and a fully connected layer is used to generate gating weights. These gating weights dynamically reflects the confidence of each branch under the current input data and can adaptively determine the weight distribution of CNN features and Transformer features in the fusion process according to the characteristics of the input audio. When the CNN features are more representative, they are given higher weights and vice versa. This dynamically adaptive fusion mechanism based on learnable weights avoids the potential information loss or redundancy issues that may arise from traditional fusion methods, allowing the model to better integrate the advantages of both architectures. The formula for generating gating weights is as follow:


$$g=\sigma (W_{g}\cdot \text{GAP}([F_{c};F_{t}^{\prime }])+b_{g}),$$ (8)

$$F_{fused}=g\cdot F_{c}+(1-g)\cdot F_{t}^{\prime }.$$ (9)

where *W*_*g*_ ∈ ${\mathbb{R}}^{1\times 2C}$ is the fully connected layer weights, g∈[0,1] dynamically reflect the confidence of each branch.

## 4. Experiments

**Fig 4:**
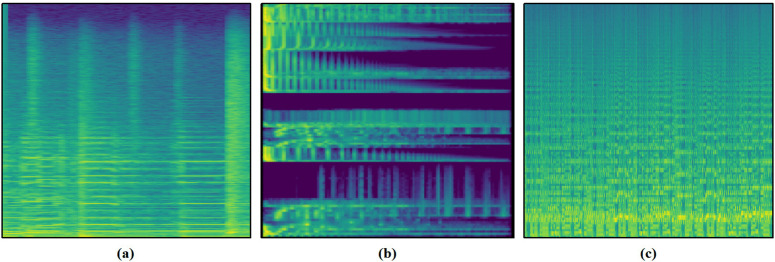
Representative spectrograms of music samples from the employed datasets: (a) Country genre from the GTZAN dataset, (b) Folk genre from the FMA-SMALL dataset, (c) Electronic genre from the FMA-Medium dataset.

This paper evaluates the performance of the proposed model in music genre classification on three publicly available datasets: GTZAN [10], FMA-SMALL and FMA-Medium [11]. Experimental results demonstrate that our approach achieves superior performance across multiple classification metrics compared to existing methods. Detailed statistics for the datasets are presented in Table 1, while Fig 4 displays representative spectrogram samples extracted from each dataset.

**Table 1:** Experimental datasets overview.

Dataset Name	Classes	Clip Length (s)	Total Clips	Class Names
GTZAN	10	30	1,000	Blues, Classical, Country, Disco, Hip-Hop, Jazz, Metal, Pop, Reggae, Rock
FMA-Small	8	30	8,000	Hip-Hop, Pop, Folk, Experimental, Rock, International, Electronic, Instrumental
FMA-Medium	16	30	25,000	Rock, Electronic, Experimental, Hip-Hop, Folk, Instrumental, Pop, International, Classical, Old-Time/Historic, Jazz, Country, Soul-RnB, Spoken, Blues, Easy Listening

### 4.1 Dataset description

This paper conducts experiments on the three datasets. The GTZAN dataset is one of the most commonly used datasets in the field of music genre classification, containing 10 genres with 100 songs per genre, each song lasting 30 seconds. These audio tracks are 22,050 Hz mono 16-bit .wav files.

Additionally, this paper utilizes the Free Music Archive (FMA), an open-source dataset. It contains 106,574 audio tracks along with their complete metadata, including song titles, album and artist information, genre tags and biographical details. The dataset is divided into three independent subsets based on scale. For this study, the small and medium-sized subsets were selected for experimentation. Specific data can be found in Table 1.

### 4.2 Data preprocessing

**Fig 5:**
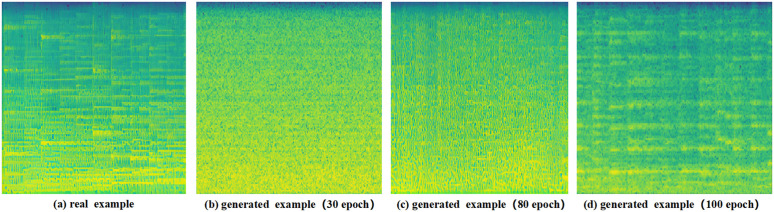
An example of the generation model on the GTZAN dataset.

Firstly, the raw waveform data were uniformly converted into mel spectrograms and the dataset was disrupted to ensure randomness. Subsequently, in order to alleviate class imbalance and enhance minority class representation, this study adopts an end-to-end audio generation method based on improved discrete cosine transform (MDCT) and denoising probabilistic probability model (DDIM). Specifically, diffusion-based audio generation is applied exclusively to underrepresented categories, while majority classes are selectively under-sampled to maintain a balanced class distribution during training. To begin with, the original audio signal is converted into real domain time-frequency representation through MDCT. The phase estimation problem in other spectrogram generation methods is avoided by using its redundancy-free and reconstructive characteristics. Then, a U-Net structure containing residual blocks and multi-head attention mechanism is designed as the core network of the diffusion model. Noise is gradually added through the forward diffusion process and the network is trained to learn the reverse denoising process. The attention mechanism is introduced in the bottleneck layer to capture long-range dependencies. In the implementation process, dynamic segmented loading and asynchronous preprocessing techniques are used to optimize the data pipeline and the cosine scheduling curve is used to control the noise addition process. Finally, efficient generation is achieved through the DDIM sampling algorithm. The pseudo code implementation is shown in Table 2. An example of the iterative process of model generation can be observed in detail by Fig 5.

**Table 2:** DDIM audio generation algorithm.

Algorithm Steps and Description	
**Input**:	Raw audio *x*, sampling rate *sr* = 10^4^, duration *T* = 3.3*s*
**Output**:	Generated audio $\hat{x}$
**Params**:	Frame size *N* = 256, steps *S*=20, U-Net widths [32,64,128,256]
**Preprocessing**	$\begin{array}{ll} X\leftarrow \text{MDCT}(x,N) & \text{(Eq.(1) time-freq transform)}\\ X_{\text{norm}}\leftarrow (X-\mu_{X})/\sigma_{X} & \text{(Standardize)} \end{array}$
**Diffusion**	Initialize U-Net *_θ_* with: *ResBlock*(width, groups = 8) ∀width ∈ widths *MultiHeadAttention*(heads = 4, key_dim = 64)
**Training**	$\begin{array}{l} \text{For}\;t∼U(1,S):\\ \;\alpha_{t}\leftarrow \cos^{2}\left(\frac{t\pi }{2S}\right)\;\text{(Cosine schedule)}\\ \;X_{t}\leftarrow \sqrt{\alpha_{t}}X_{\text{norm}}+\sqrt{1-\alpha_{t}}\varepsilon \\ \;L\leftarrow {∥\varepsilon -\varepsilon_{\theta }(X_{t},t)∥}_{2}^{2}+\lambda {∥\nabla_{t}X-\nabla_{t}\hat{X}∥}_{1} \end{array}$
**Generation**	$\begin{array}{l} X_{S}∼N(0,I)\;\text{(Initialize noise)}\\ \text{For }t=S\text{ downto }1:\\ \;X_{t-1}\leftarrow \frac{1}{\sqrt{\alpha_{t}}}\left(X_{t}-\frac{1-\alpha_{t}}{\sqrt{1-{\bar{\alpha }}_{t}}}\hat{\varepsilon }\right)\\ \hat{x}\leftarrow \text{iMDCT}(\sigma_{X}X_{0}+\mu_{X})\;\text{(Reconstruct)} \end{array}$

^1^Key symbols: ${\bar{\alpha }}_{t}=\prod_{s=1}^{t}\alpha_{s}$, $\nabla_{t}$: temporal difference operator.

### 4.3 Evaluation metrics

In order to measure the model performance, this paper evaluates the performance of the music genre classification system using four standard metrics: precision, recall, F1 score and accuracy. The experiments were performed on three widely-used datasets: GTZAN, FMA-SMALL and FMA-Medium.

### 4.4 Experimental settings

To ensure statistical reliability of experimental results, all performance metrics were calculated using 10-fold cross-validation, with final reported values representing the average performance across all music genres. All datasets were evenly split into training, validation and test sets at an 8:1:1 ratio. This model employs a hybrid backbone architecture followed by a classification head with a softmax output, totaling 9 million parameters. The proposed model was implemented using the TensorFlow 2.6 framework, trained with the Nadam optimizer and a fixed learning rate of 0.001. The training objective employed a labeled-smoothed classification cross-entropy loss with a smoothing coefficient of 0.1 to enhance model calibration and improve generalization performance. Customized training configurations were applied for datasets of varying feature scales. The GTZAN dataset underwent 100 training epochs with a batch size of 32. To maintain experimental fairness, the large-scale FMA dataset was trained for 80 epochs using the same batch size. All training processes implemented an early stopping strategy with a patience threshold of 15 epochs, using validation set accuracy as the monitoring metric to effectively suppress overfitting. To address the inherent class imbalance in the dataset, a focus loss with class-dependent weights was employed during training. Additionally, a hybrid data balancing strategy was implemented, involving downsampling of the majority class and diffusion-based data generation techniques for the minority class. All experiments were conducted on a hardware platform equipped with an Intel Core i5-12400 processor and NVIDIA GeForce RTX 4060 GPU, utilizing GPU acceleration.

## 5. Results and analysis

This section presents a comprehensive evaluation of the proposed CT-GateNet model on three widely used public music genre classification benchmarks, namely GTZAN, FMA-SMALL and FMA-Medium. By comparing the proposed approach with representative state-of-the-art methods as well as mainstream deep learning backbones, the effectiveness, robustness and generalization capability of CT-GateNet are systematically validated.

### 5.1 Results on the GTZAN dataset

The GTZAN dataset is first employed to evaluate the classification performance of CT-GateNet under a relatively balanced and well-studied benchmark setting. Quantitative comparisons with representative state-of-the-art approaches are summarized in Table 3. As shown, CT-GateNet achieves an overall classification accuracy of 98.72%, significantly outperforming existing methods by a clear margin. In addition to accuracy, the proposed model also achieves consistently high precision (98.75%), recall (98.72%) and F1-score (98.73%), indicating a well-balanced and reliable classification performance across all music genres.

**Table 3:** Performance comparison with state-of-the-art methods on GTZAN dataset.

Model	Accuracy (%)	Precision (%)	Recall (%)	F1 (%)	#Params
Heakl et al. [4]	70.00	–	–	–	–
Jena et al. [12]	80.40	81.32	80.10	80.20	–
Medhat et al. [13]	85.10	85.68	85.19	84.92	–
M. Ashraf et al. [14]	86.00	–	–	–	–
Chen et al. [5]	87.41	87.93	87.58	87.28	–
N. Farajzadeh et al. [15]	87.80	92.67	88.20	90.38	–
Ashraf et al. [16]	89.30	85.00	–	88.00	**2.4M**
Ahmed et al. [17]	92.71	93.00	–	96.00	6.5M
Prabhakar et al. [18]	93.51	–	–	–	8.6M
Dwivedi et al. [19]	98.97	–	–	–	4.2M
**CT-GateNet (Ours)**	**98.72**	**98.75**	**98.72**	**98.73**	9M

To further analyze the effectiveness of the proposed architecture, Table 4 compares CT-GateNet with several representative deep learning backbones, including lightweight CNNs, deeper convolutional networks and Transformer-based models. Among these baselines, DenseNet121 achieves the highest accuracy 86.31% among traditional CNN architectures, while pure Transformer-based models such as ViT Small exhibit substantially lower performance 71.03%. These results highlight the importance of effective local feature modeling in spectrogram-based music genre classification. Although ConvNeXt incorporates several Transformer-inspired design principles, it remains a convolutional architecture and still falls short of deeper CNN backbones, indicating that architectural design alone is insufficient without task-specific feature fusion mechanisms.

**Table 4:** Performance comparison of deep learning backbones and proposed method on GTZAN dataset.

Model	Accuracy (%)	Precision (%)	Recall (%)	F1 (%)
MobileNet V3 Small	79.88	79.68	80.00	79.43
MobileNet V3 Large	78.91	78.64	78.70	79.70
EfficientNet	78.42	77.94	78.20	77.34
DenseNet121	86.31	86.88	86.40	85.92
ResNet34	84.55	84.71	84.30	83.80
Inception V4	82.03	82.48	81.90	81.73
ConvNeXt	81.04	80.67	80.49	80.16
ViT Small	71.03	71.62	71.10	70.46
MobileViT Small	73.02	74.04	73.00	72.97
**CT-GateNet (Ours)**	**98.72**	**98.75**	**98.72**	**98.73**

**Fig 6:**
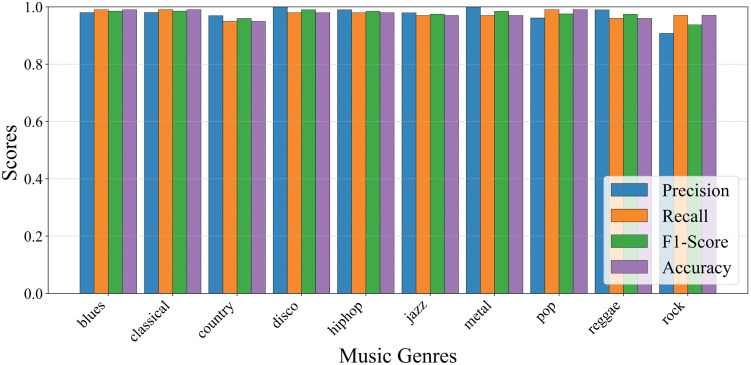
Performance metrics of each target music genre on GTZAN dataset.

Fig 6 illustrates the precision, recall, F1-score and accuracy achieved by CT-GateNet on three representative music genres Classical, Country and Hip-Hop. All metrics remain consistently above 0.8 with limited variance, demonstrating the model’s stable discriminative capability across different genre characteristics. This observation aligns well with the overall quantitative results reported in Table 4.

**Fig 7:**
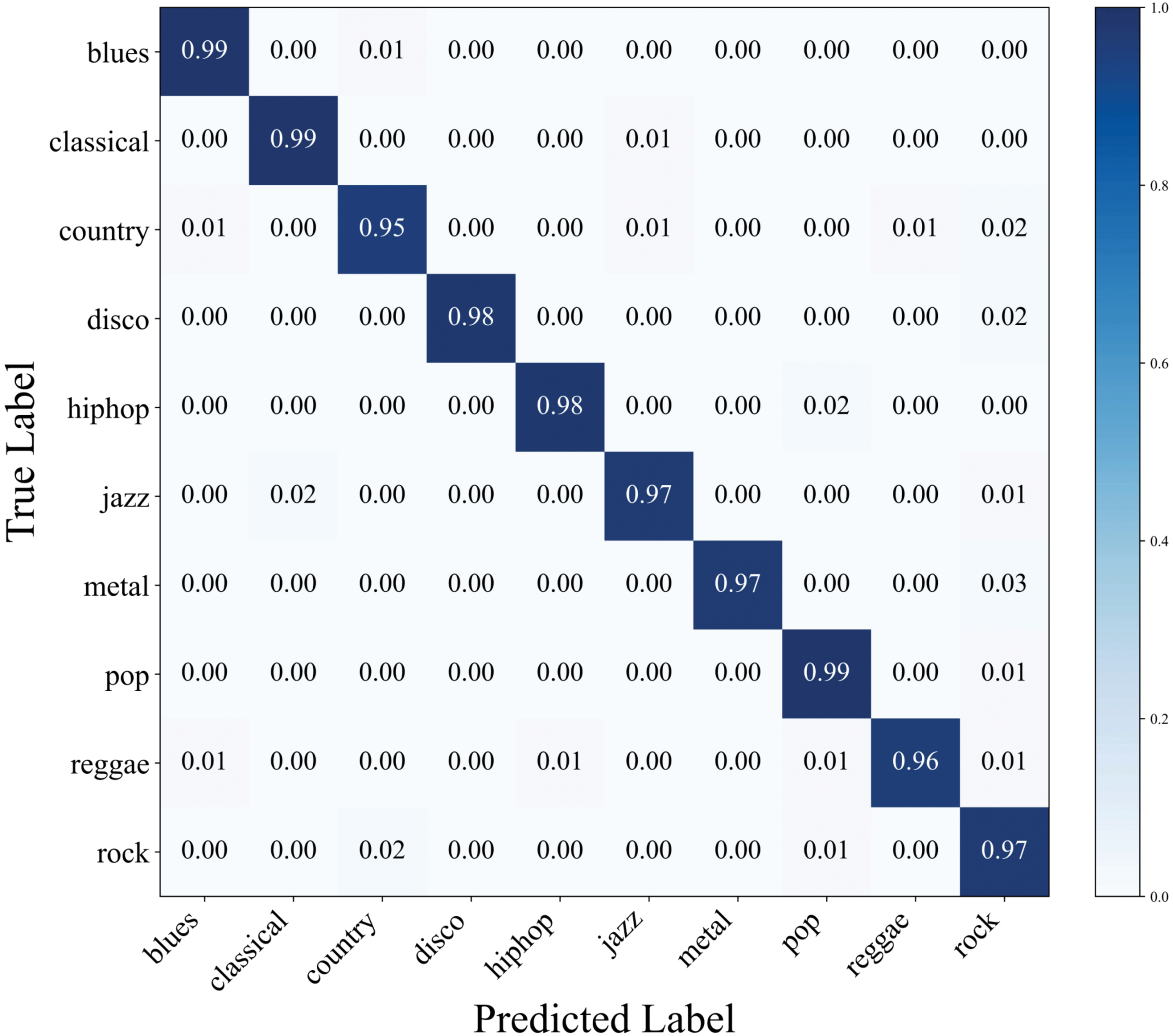
GTZAN normalized confusion matrix (test set).

Fig 7 shows the normalized confusion matrix of CT-GateNet on the GTZAN test set. The matrix exhibits a clear dominance of diagonal elements, with most values above 0.95, indicating that the model achieves excellent classification performance across all ten genres. Minor misclassifications are limited and primarily occur between acoustically related genres, such as a small confusion between Country and Rock, and between Reggae and Rock. Overall, the sparse off-diagonal entries demonstrate that CT-GateNet effectively learns discriminative genre-specific features while maintaining strong robustness against inter-genre similarity.

Notably, a recent study by Dwivedi et al. [19] achieved an overall classification accuracy of 98.97% on the GTZAN dataset, slightly higher than the 98.72% attained by the CT-GateNet model proposed in this paper. However, a detailed analysis of their published category-specific results reveals certain differences between the two approaches. Despite its higher overall accuracy, their model exhibits relatively inconsistent performance across various music genres. For instance, its F1 score for the Reggae genre is only 0.84, and its precision for Jazz is approximately 86%. In contrast, the proposed CT-GateNet demonstrates stable and outstanding performance across all ten music genres. As shown in Fig 6, our model consistently maintains scores above 0.90 across all categories under the same classification metrics. These results demonstrate that CT-GateNet effectively mitigates performance imbalances between genres while maintaining competitive overall accuracy. This balanced single-category performance is particularly desirable for practical music genre classification systems, as it indicates the model not only optimizes overall accuracy but also enhances robustness and generalization capabilities.

### 5.2 Results on the FMA-SMALL dataset

On the FMA-SMALL dataset, the comparative experiments with existing methods presented in Table 5, CT-GateNet achieved a classification accuracy of 89.42%, outperforming the methods proposed by Zhang et al., N. Farajzadeh et al., and J. Shen et al., while only slightly falling short of the results by P. Dwivedi et al. Additionally, its parameter size of 9 million remains within a reasonable range. As shown in Table 6, CT-GateNet achieves accuracy rates that are more than ten percentage points higher than ViT Small, MobileNet V3 Small and EfficientNet V2.

**Table 5:** Performance comparison with state-of-the-art methods on FMA-SMALL dataset.

Model	Accuracy (%)	Precision (%)	Recall (%)	F1 (%)	#Params
Zhang et al. [20]	64.70	–	43.50	42.30	**0.5M**
N. Farajzadeh et al. [15]	68.80	66.30	61.70	63.92	5M
J. Shen et al. [21]	88.60	88.30	88.80	88.50	–
P. Dwivedi et al. [19]	**92.0**	88.0	89.0	88.0	4.5M
**CT-GateNet (Ours)**	89.42	**89.51**	**89.42**	**89.31**	9M

**Table 6:** Performance comparison of deep learning backbones and proposed method on FMA-SMALL dataset.

Model	Accuracy (%)	Precision (%)	Recall (%)	F1 (%)
ViT Small	78.65	78.20	78.65	78.42
MobileNet V3 Small	80.12	79.85	80.12	79.98
EfficientNet V2	82.85	82.50	82.85	82.67
**CT-GateNet (Ours)**	**89.42**	**89.51**	**89.42**	**89.31**

**Fig 8:**
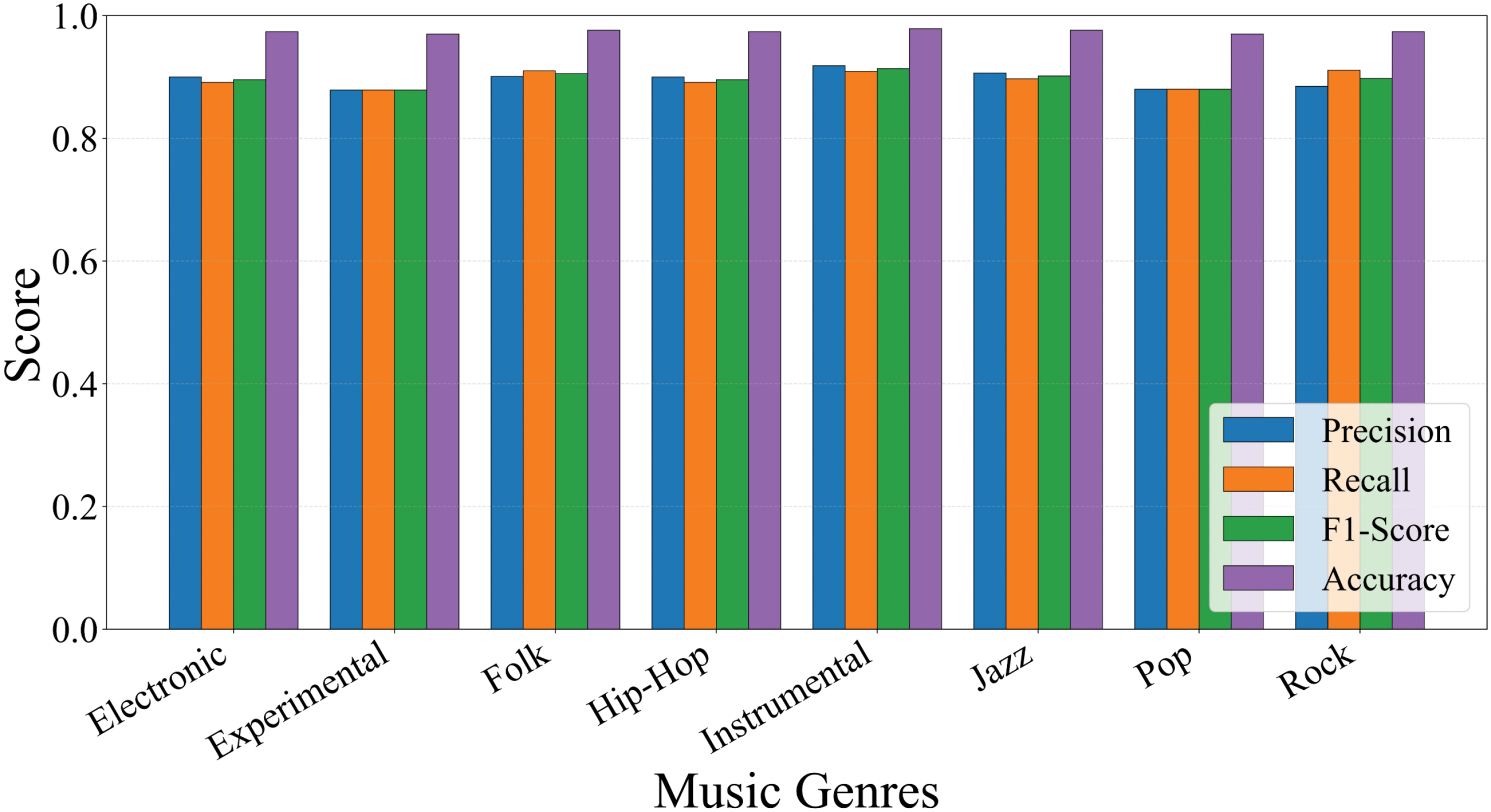
Performance metrics of each target music genre on FMA-SMALL dataset.

**Fig 9:**
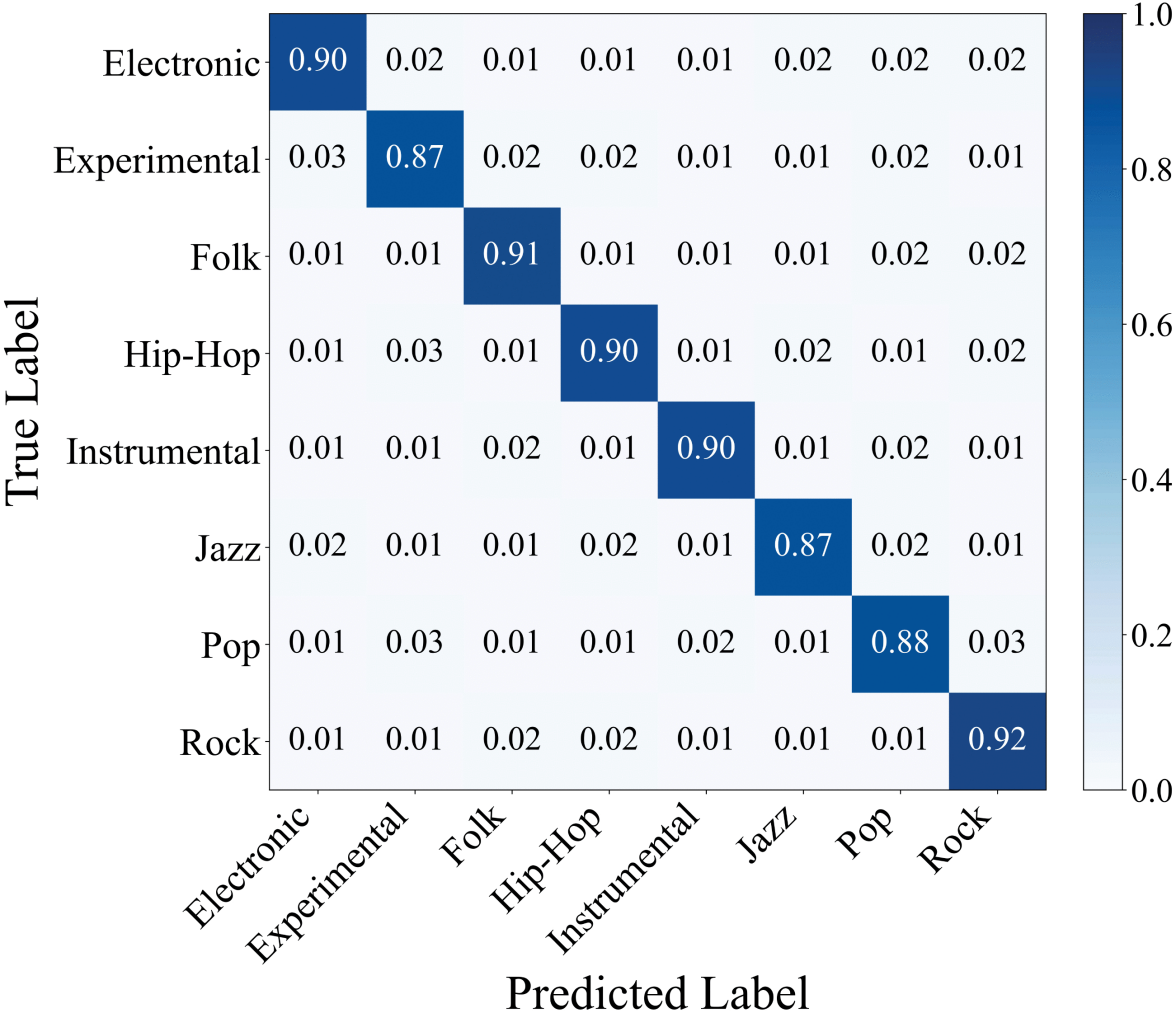
FMA-SMALL normalized confusion matrix (test set).

In Fig 8 indicates that precision, recall and F1 scores for each genre remain stable within the ranges of 0.91 to 0.93, 0.87 to 0.92, and 0.89 to 0.92, respectively, all exceeding 0.87. The overall micro-averaged precision, recall and F1 scores are 89.51%, 89.42% and 89.31%, respectively. Fig 9 shows that the model achieves recall above 0.87 across all 8 music genres. Recall rates for rock, folk, hip-hop, electronic and instrumental genres reached 0.92, 0.91, 0.90, 0.90, and 0.90 respectively, demonstrating particularly strong performance. Recall rates for pop, jazz, and experimental genres were 0.88, 0.87, and 0.87, maintaining similarly high levels.

Experimental results demonstrate that CT-GateNet can clearly distinguish the characteristics of different music genres without producing widespread cross-category misclassifications. The significant improvement in pop genre recognition performance proves that the model effectively addresses the classification challenges posed by this genre’s high stylistic fusion and blurred feature boundaries. Balanced performance across genres reflects the model’s strong generalization capability for diverse musical styles, avoiding overfitting to any single type.

### 5.3 Results on the FMA-Medium dataset

To further evaluate the scalability and robustness of the proposed model under more challenging and realistic conditions, experiments are conducted on the FMA-Medium dataset. Compared to GTZAN, FMA-Medium contains a substantially larger number of tracks spanning 16 music genres and exhibits a pronounced long-tail distribution, with significant imbalance among genre categories. This dataset therefore provides a more demanding benchmark for evaluating model generalization.

Table 7 compares CT-GateNet with representative state-of-the-art methods on the FMA-Medium dataset. While most existing studies primarily report overall classification accuracy, CT-GateNet achieves a competitive accuracy of 68.50%, outperforming the recent method by Ding et al. [24] by 0.43 percentage points. More importantly, the proposed method reports comprehensive multi-class evaluation metrics, including precision 69.20%, recall 68.50% and F1-score 68.80%, enabling a more thorough and fair assessment of classification performance on imbalanced data.

**Table 7:** Performance comparison with state-of-the-art methods on FMA-Medium dataset.

Model	Accuracy (%)	Precision (%)	Recall (%)	F1 (%)	#Params
Kim et al. [22]	66.29	–	–	–	4.7M
Lee et al. [23]	62.50	–	–	–	**1.64M**
Ding et al. [24]	68.07	–	–	–	8.6M
**CT-GateNet (Ours)**	**68.50**	**69.20**	**68.50**	**68.80**	9M

Further comparisons with mainstream deep learning backbones are provided in Table 8. The evaluated backbones, including ViT Small, MobileNet V3 Small, EfficientNet V2 and ResNet50, achieve accuracy values ranging from 64.75% to 66.94%. In contrast, CT-GateNet consistently outperforms all baseline models across all evaluation metrics, confirming the effectiveness of the proposed fusion architecture in handling complex and diverse music genre distributions.

**Table 8:** Performance comparison of deep learning backbones and proposed method on FMA-Medium dataset.

Model	Accuracy (%)	Precision (%)	Recall (%)	F1 (%)
ViT Small	64.75	62.63	64.75	63.67
MobileNet V3 Small	65.32	63.25	65.32	64.27
EfficientNet V2	66.18	64.36	66.18	65.25
ResNet50	66.94	65.12	66.94	66.02
**CT-GateNet (Ours)**	**68.50**	**69.20**	**68.50**	**68.80**

**Fig 10:**
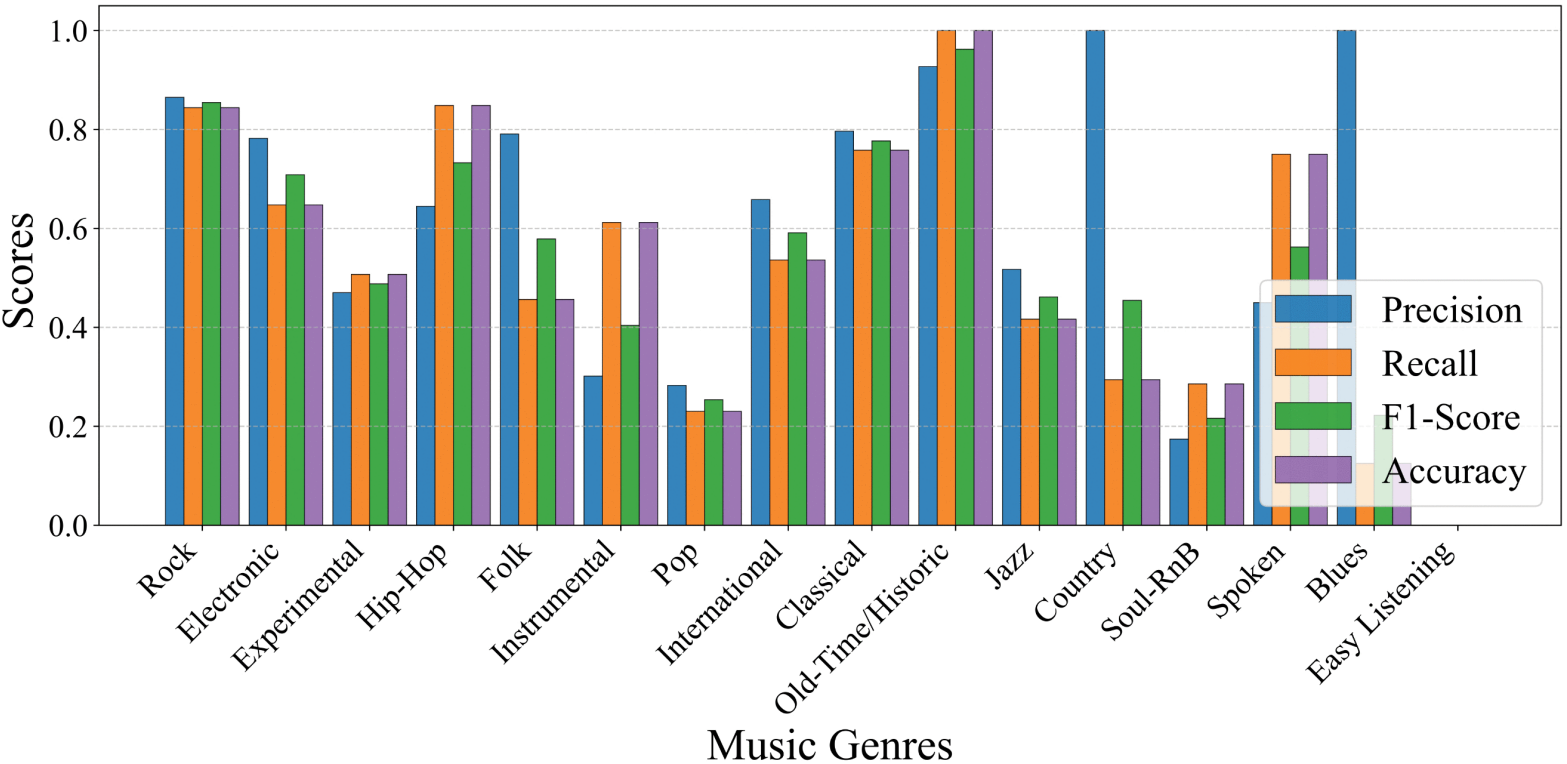
Performance metrics of each target music genre on FMA-Medium dataset.

Fig 10 visualizes the per-class classification performance of CT-GateNet across all 16 genres in the FMA-Medium dataset. The model achieves strong performance for majority genres with abundant training samples, such as Rock, Old-Time/Historic and Spoken. However, performance degradation is observed for severely underrepresented genres, including Blues and Easy Listening, reflecting the inherent challenges posed by extreme class imbalance.

**Fig 11:**
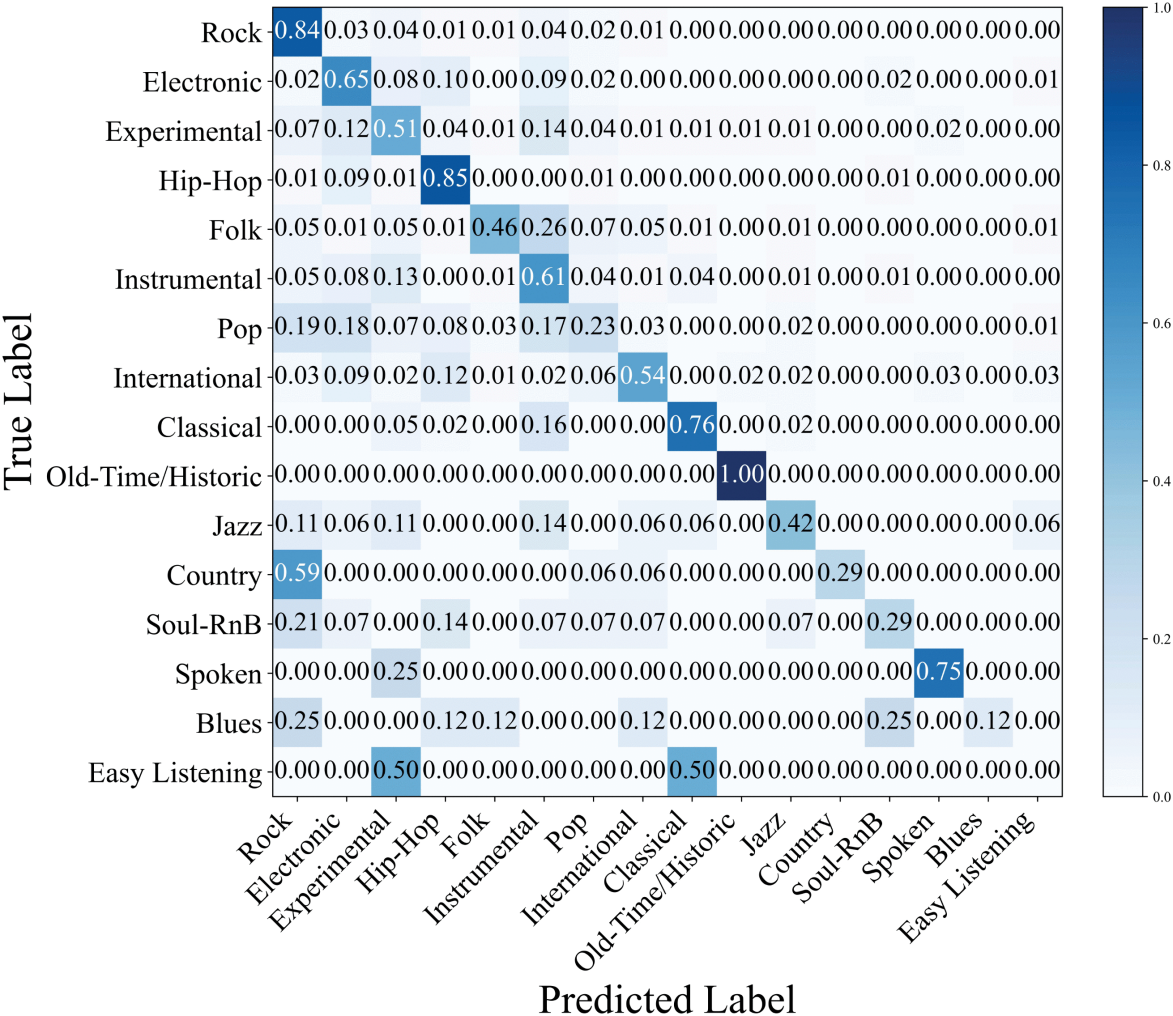
FMA-Medium normalized confusion matrix (test set).

The normalized confusion matrix in Fig 11 shows that CT-GateNet achieves high performance on genres with distinctive acoustic characteristics while struggling with categories that exhibit stylistic overlap. Old-Time/Historic attains perfect classification accuracy, with no confusion with other genres. Rock and Hip-Hop achieve high accuracies of 0.84 and 0.85, with only minor misclassifications such as Rock being occasionally identified as Electronic. Classical and Folk exhibit moderate confusion due to shared acoustic instrumentation, and Spoken and Experimental overlap because of similar vocal timbres. More substantial errors arise from stylistic fusion: Country is often misclassified as Rock, leading to an accuracy of 0.29; Pop is frequently confused with Rock and Electronic, achieving 0.23 accuracy; Blues is divided between Rock and Soul-RnB, resulting in 0.25 correct classification; Easy Listening is commonly misclassified as Classical, yielding 0.50 accuracy. These patterns indicate that CT-GateNet excels with acoustically distinct genres but faces limitations when styles are highly overlapping or hybrid.

Experimental results combining the FMA-SMALL and FMA-MEDIUM datasets demonstrate that the proposed model not only achieves outstanding performance in balanced data scenarios but also outperforms existing methods under conditions of severe data imbalance.

### 5.4 Impact of class imbalance

Although CT-GateNet achieves competitive overall accuracy on the FMA-Medium dataset, a clear gap remains between accuracy and macro-averaged F1-score, primarily due to the dataset’s severe class imbalance. As shown in Fig 10 and 11, majority genres such as Rock, Electronic and Hip-Hop obtain higher recall and thus dominate the overall accuracy, whereas minority genres with limited samples are more prone to misclassification, resulting in reduced per-class recall and F1-scores. This observation indicates that overall accuracy alone is insufficient for evaluating performance on long-tail music genre datasets, and macro-level metrics are necessary to reflect balanced classification capability.

To alleviate class imbalance during training while maintaining realistic evaluation conditions, a hybrid data balancing strategy was adopted. Specifically, random undersampling was applied to majority classes, while a diffusion-based generative model was used to augment minority classes. This strategy was applied exclusively to the training set, whereas the validation and test sets preserved the original long-tail distribution of FMA-Medium. As a result, the reported performance reflects genuine generalization ability under real-world imbalanced conditions rather than artificially balanced evaluation data. Future work will investigate more advanced imbalance-aware learning strategies to further enhance minority genre recognition.

In summary, CT-GateNet comprises approximately 9 million parameters, maintaining comparable levels to recent state-of-the-art models such as Ding et al.’s 8.6-million-parameter model and Ahmed et al.’s 6.5-million-parameter model. With its moderate model size, CT-GateNet demonstrates competitive classification performance across public benchmarks while maintaining stable cross-class performance under class imbalance conditions.

## 6. Discussion

To further validate the effectiveness and generalization capability of the proposed CT-GateNet architecture, a series of ablation experiments were conducted on the GTZAN, FMA-SMALL and FMA-Medium datasets, with the results summarized in Tables 9–11. This dual-dataset ablation design ensures that the effectiveness of each architectural module is not confined to a single benchmark: the GTZAN dataset provides a well-balanced experimental setting to quantify the pure contribution of each component to classification performance, while the FMA-Medium dataset validates the robustness of these modules on a large-scale, real-world imbalanced dataset with more complex genre distributions.

**Table 9:** The ablation experiment results on the GTZAN dataset.

Model	Accuracy (%)	Precision (%)	Recall (%)	F1 (%)
No GCSA module	94.57	94.75	94.57	94.59
No AFFG module	96.16	96.21	96.16	96.16
No CNN module	92.40	92.49	92.40	92.33
No Transformer encoder and AFFG module	95.58	95.76	95.58	95.62
**CT-GateNet (Ours)**	**98.72**	**98.75**	**98.72**	**98.73**

**Table 10:** The ablation experiment results on the FMA-SMALL dataset.

Model	Accuracy (%)	Precision (%)	Recall (%)	F1 (%)
No GCSA module	84.12	84.05	84.12	84.08
No AFFG module	82.37	82.29	82.37	82.33
No CNN module	79.65	79.58	79.65	79.61
No Transformer encoder				
and AFFG module	76.82	76.75	76.82	76.78
**CT-GateNet (Ours)**	**89.42**	**89.51**	**89.42**	**89.31**

**Table 11:** The ablation experiment results on the FMA-Medium dataset.

Model	Accuracy (%)	Precision (%)	Recall (%)	F1 (%)
No GCSA module	65.38	65.92	65.38	65.57
No AFFG module	66.42	67.15	66.42	66.74
No CNN module	61.22	61.89	61.22	61.53
No Transformer encoder				
and AFFG module	64.45	65.07	64.45	64.72
**CT-GateNet (Ours)**	**68.50**	**69.20**	**68.50**	**68.80**

In these ablation settings, “No GCSA module” denotes the removal of the GCSA module from the architecture. “No AFFG module” indicates that the AFFG module is excluded and the CNN and Transformer features are manually fused using fixed weights of 0.6 and 0.4, respectively. “No CNN module” refers to removing the CNN branch, and “No Transformer encoder and AFFG module” indicates the removal of both the Transformer encoder and the AFFG module. As shown in Table 9, removing the GCSA module leads to a 4.15% drop in classification accuracy on the GTZAN dataset, demonstrating the importance of enhanced local feature modeling. Excluding the AFFG module also results in noticeable performance degradation, indicating that adaptive feature fusion is more effective than fixed-weight fusion. The most significant performance decline is observed when the CNN module is removed, highlighting the critical role of local time-frequency feature extraction in spectrogram-based music genre classification. In addition, removing the Transformer encoder causes a 3.14% reduction in accuracy, suggesting that long-range dependency modeling contributes complementary global information to the overall performance.

Ablation experiments on the FMA-SMALL dataset, as shown in Table 10, demonstrate that removing the GCSA module, AFFG module, CNN module and simultaneously removing the Transformer encoder and AFFG module results in a progressive decline in the model’s accuracy, precision, recall and F1-score. The complete CT-GateNet model achieves optimal performance, which fully validates the critical role of each core component in the model’s feature extraction and classification capabilities.

The ablation results on the FMA-Medium dataset, as shown in Table 11, exhibit consistent trends with those on GTZAN, which further validates the universality and effectiveness of each designed module rather than dataset-specific performance gains. On the FMA-Medium dataset, removing the GCSA module leads to a 3.12% accuracy drop, confirming that the GCSA mechanism stably enhances the extraction of discriminative local time-frequency features regardless of dataset scale and class distribution. Excluding the AFFG module causes a 2.08% accuracy reduction, which again proves that adaptive gated feature fusion outperforms static fixed-weight fusion—this advantage is even more pronounced on the imbalanced FMA-Medium dataset, as adaptive fusion can dynamically adjust feature weights to focus on minority genre features with sparse acoustic representations.

The most significant performance degradation is also observed when the CNN module is removed, a 7.28% accuracy drop on FMA-Medium, which is consistent with the GTZAN results. This phenomenon fully illustrates that convolutional-based local feature modeling is the cornerstone of spectrogram-based music genre classification: the CNN branch captures fine-grained frequency band correlations and short-term temporal patterns that are irreplaceable for distinguishing music genres, especially for minority genres with unique acoustic signatures. Removing the Transformer encoder and AFFG module results in a 4.05% accuracy drop, indicating that the Transformer’s long-range dependency modeling can effectively capture the global structural information of audio spectrograms, which compensates for the CNN’s deficiency in modeling long-sequence temporal correlations and further improves classification performance on large-scale datasets.

To comprehensively evaluate the effectiveness of the DDIM-based data augmentation scheme, this paper systematically compares two baseline approaches:

**No-augmentation strategy**: Training directly on the original GTZAN dataset without any data augmentation techniques, serving as the performance lower bound benchmark.
**Traditional audio augmentation**: Segmenting audio into four-second clips, with fifty percent of the clips randomly subjected to time stretching or pitch shifting transformations—time stretching applies ±10% rate variation, pitch shifting adjusts by ±1 semitone, and both transformations are simultaneously applied with a twenty percent probability. The augmented data is subsequently merged with the original data to form the training set, representing the performance level of current mainstream augmentation methods.

The proposed DDIM-based enhancement scheme is detailed in Table 2. All experiments adopt identical model architectures, training procedures, and hyperparameter settings to ensure fair comparison. Experimental results on both GTZAN and FMA-SMALL datasets are presented in Table 12. Our DDIM-based augmentation achieves the highest performance on both datasets: 98.72% accuracy on GTZAN and 89.42% accuracy on FMA-SMALL. Notably, the performance gap between different augmentation methods is much larger on FMA-SMALL than on GTZAN. This is because GTZAN has a simpler data composition, making traditional augmentation methods slightly effective, while FMA-SMALL has higher data complexity, where traditional augmentation yields limited improvements. In contrast, our DDIM-based method generates samples with higher semantic authenticity, effectively capturing key data distribution characteristics and thus achieving more significant performance gains, especially on complex datasets.

**Table 12:** Performance comparison of different data augmentation methods on GTZAN and FMA-SMALL datasets.

Data Augmentation Method	GTZAN (%)	FMA-SMALL (%)
No Augmentation	95.93	84.50
Traditional Audio Augmentation	96.66	86.20
DDIM-based Augmentation	**98.72**	**89.42**

Based on the experimental results in Section 5, the proposed model outperforms existing mainstream approaches in performance. The main advantages originate from the following design features. First, the hybrid architecture captures both local features and long-range dependencies. Second, in consideration of the limited scale of the music dataset, a shallow structure—comprising only 5 CNN layers and 2 Transformer encoder layers—is adopted, thereby effectively mitigating the risk of overfitting. Experiments on Deep Learning Backbones demonstrate that deeper networks do not necessarily lead to better performance in this task. Furthermore, the improved GCSA mechanism enhances local feature extraction, while the AFFG mechanism facilitates adaptive fusion of cross-layer features. As shown in Tables 9 and 12, both components contribute significantly to performance improvement on both datasets. However, this study has certain limitations. First, due to hardware constraints, validation on large-scale datasets was not conducted. Second, the time-consuming nature of sample generation in the model restricts the scale of augmented data. Future work will focus on optimizing generation efficiency and developing more effective data augmentation strategies by drawing on methods such as those reported in [25] and [26].

## 7. Conclusions

In this paper, a hybrid CNN and Transformer model structure, CT-GateNet, is proposed for the music genre classification task. To address the problem of weak local feature extraction ability during model training, the GCSA mechanism is proposed to significantly improve the local feature extraction ability of the model. For global feature extraction, a Transformer encoder is combined to capture long-range dependencies. To overcome the need for manual feature fusion in hybrid architectures, the proposed AFFG mechanism automatically and effectively integrates features from CNN and Transformer components, eliminating the cumbersome process of manual weight tuning and optimal weight selection. Experimental results demonstrate that CT-GateNet effectively improves the accuracy of music classification and achieves competitive performance on the GTZAN, FMA-SMALL and FMA-Medium datasets.
